# Insight from the microelectrodes in case of two different types of premature ventricular contractions originating from left ventricular summit

**DOI:** 10.1016/j.ipej.2024.05.001

**Published:** 2024-05-08

**Authors:** Shushi Nishiwaki, Satoshi Shizuta, Hirohiko Kohjitani, Koh Ono

**Affiliations:** Department of Cardiovascular Medicine, Kyoto University Graduate School of Medicine, Kyoto, Japan

**Keywords:** Microelectrodes, Premature ventricular contraction, Left ventricular summit, Left coronary cusp, Great cardiac vein

## Abstract

Premature ventricular contraction (PVC) is usually eliminated in the earliest activation site based on the conventional electrode of ablation catheter. However, the large size electrode may contain far-field potential. The QDOT MICRO ablation catheter has three micro electrodes with 0.33 mm electrode length, in addition to the conventional electrode with 3.5 mm electrode length. The micro electrodes can reflect only near-field potential.

A 78-year-old with symptomatic frequent PVCs underwent catheter ablation. PVC-1 showed good pace-mapping in distal great cardiac vein (GCV). The local bipolar electrograms in the conventional electrode of ablation catheter preceded the PVC-QRS onset by 32 ms in distal GCV and 13 ms in left coronary cusp (LCC), but those in the micro electrodes preceded only by 13 ms both in distal GCV and LCC. PVC-1 was eliminated by radiofrequency (RF) application, not in distal GCV, but in LCC. PVC-2 showed good pace-mapping in LCC. The local bipolar electrograms in both the conventional electrode and the micro electrodes of ablation catheter preceded the PVC-QRS onset by 32 ms in LCC. PVC-2 was eliminated by RF application in LCC.

Comparing the local electrograms of micro electrodes and the conventional electrodes may be important for identifying depth of the origin of PVCs.

## 1 Introduction

Premature ventricular contraction (PVC) originating from left ventricular (LV) summit, highest point of the LV, remains a challenge for catheter ablation. Several ablation techniques have been proposed from accessible adjacent sites, including the right ventricular outflow tract (RVOT), the left coronary cusp (LCC), the LV endocardium, and the coronary venous system [1]. In these techniques for the LV summit PVC, pace-mapping is less important owing to preferential conduction and the proximity of multiple structures. Therefore, the key to successful ablation is searching for the earliest activation site using conventional electrodes of ablation catheter. However, the conventional electrodes of ablation catheter may contain far-field potential, because the conventional electrodes are larger than micro electrodes of multipolar mapping catheter [2]. Accordingly, the ablation for PVC is not always successful in the earliest activation site of conventional electrodes of ablation catheter. We present a case of two different types of PVCs originating from LV summit simultaneously eliminated by catheter ablation for LCC using micro electrode-embedded catheter.

## 2 Case report

**Fig. 1:**
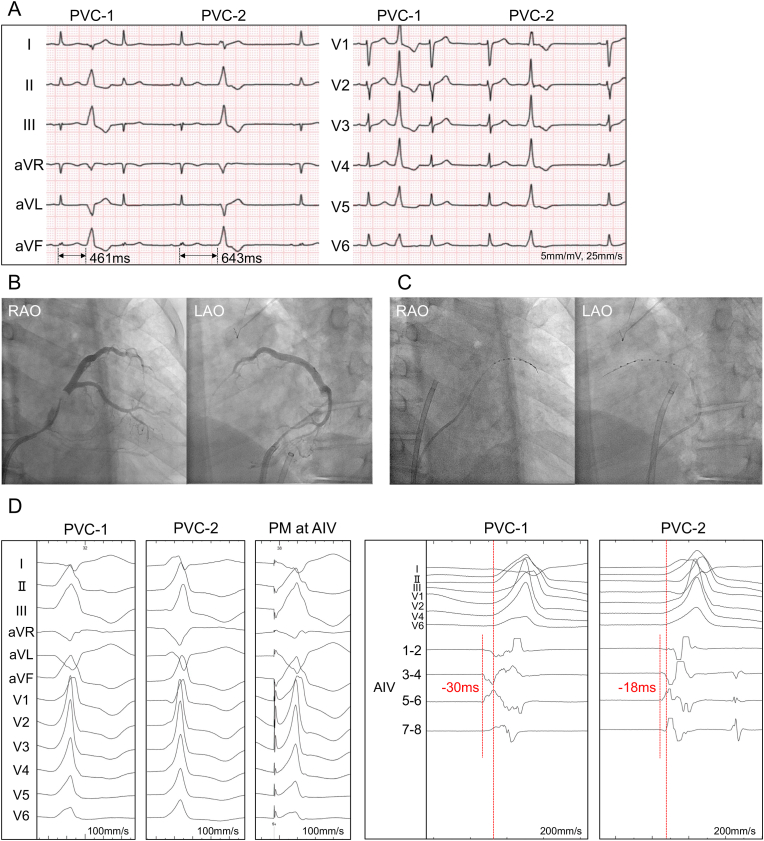
(A) Twelve-lead ECG of clinical PVCs. Coupling interval was shorter in PVC-1 (461 ms) than in PVC-2 (643 ms). (B) The fluoroscopic images of retrograde coronary sinus venography. (C) The fluoroscopic images of the placement of electrode catheter in AIV and GCV. (D) Twelve-lead ECG of PVC-1, PVC-2 and pace-mapping at proximal AIV (AIV 3–4) with good score of 98 % for PVC-1 (left). The intracardiac ECG recordings of PVC-1 and PVC-2 (right). The electrograms of AIV 3–4 preceded the PVC-QRS onset by 30 ms in PVC-1 and 18 ms in PVC-2. PVC, premature ventricular contraction; RAO, right anterior oblique; LAO, left anterior oblique; PM, pace-mapping; AIV, anterior interventricular vein; GCV, great cardiac vein; ECG, electrocardiogram.

A 78-year-old man with no structural heart disease experienced frequent palpitations. A 24-h Holter monitoring recorded a total of 8017 PVCs per day (9 %). Twelve-lead electrocardiogram showed two distinct types of PVCs with an inferior axis and right bundle branch block morphology (Fig. 1A). PVC-1 had a short coupling interval of 461 ms and QS pattern in lead I, while PVC-2 had a long coupling interval of 643 ms and RS pattern in lead I. He underwent catheter ablation for PVCs. Retrograde coronary sinus venography showed no communicating veins (Fig. 1B). A 3 Fr octopolar monorail-type catheter with 1.0 mm electrode length and 5.0 mm interelectrode spacing (InterNova Monorail II+, InterNova, Tokyo, Japan) was introduced into the anterior interventricular vein (AIV) and the great cardiac vein (GCV) with a 0.014 inch guidewire (Fig. 1C). In both PVC-1 and PVC-2, the earliest activation site was the proximal AIV (AIV 3–4), preceding the PVC-QRS onset by 30 ms in PVC-1 and 18 ms in PVC-2 (Fig. 1D). Pace-mapping at the AIV 3–4 was good for PVC-1 with a score of 98 % in the electrophysiology recording system (LabSystem Pro, Boston Scientific, Natick, MA, USA) (Fig. 1D). Thus, the origin of PVC-1 was consitdered to be near the proximal AIV.

**Fig. 2:**
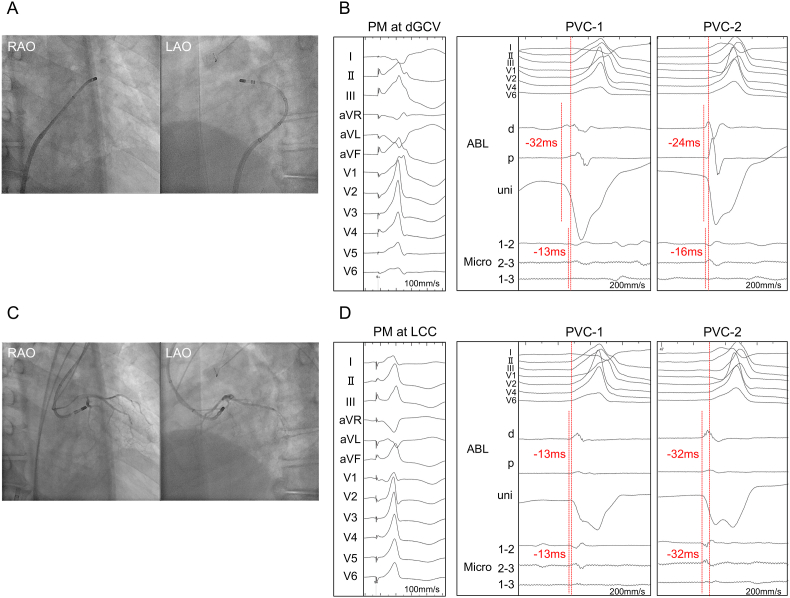
(A)The fluoroscopic images of the placement of ablation catheter in distal GCV. (B) Twelve-lead ECG of pace-mapping at distal GCV with good score of 94 % for PVC-1 (left). The intracardiac ECG recordings of PVC-1 and PVC-2 (right). The electrograms of conventional electrode (ABL d) preceded the PVC-QRS onset by 32 ms in PVC-1 and 24 ms in PVC-2. The electrograms of the microelectrodes (Micro 1–2 and 2–3) preceded the PVC-QRS onset by 13 ms in PVC-1 and 16 ms in PVC-2. (C)The fluoroscopic images of the placement of ablation catheter in LCC, and angiography of left coronary artery. (D)Twelve-lead ECG of pace-mapping at LCC with good score of 92 % for PVC-2 (left). The intracardiac ECG recordings of PVC-1 and PVC-2 (right). The electrograms of both conventional (ABL d) and microelectrodes (Micro 1–2 and 2–3) preceded the PVC-QRS onset by 13 ms in PVC-1 and 32 ms in PVC-2. RAO, right anterior oblique; LAO, left anterior oblique; PM, pace-mapping; d, distal; GCV, great cardiac vein; PVC, premature ventricular contraction; ABL, ablation catheter; p, proximal; uni, unipolar recording; LCC, left coronary cusp; ECG, electrocardiogram.

An 8 Fr 3.5 mm irrigated-tip ablation catheter with three embedded microelectrodes (QDOT MICRO bi-directional D/F-curve, Biosense Webster, Diamond Bar, CA, USA) was placed into distal GCV (Fig. 2A). In PVC-1, the electrograms of ablation catheter preceded the PVC-QRS onset by 32 ms in the conventional electrode and 13 ms in microelectrodes (Fig. 2B). In PVC-2, the electrograms of ablation catheter preceded the PVC-QRS onset by 24 ms in the conventional electrode and 13 ms in microelectrodes. Pace-mapping at distal electrodes of ablation catheter was also good for PVC-1 with a score of 94 % (Fig. 2B). Based on the electrogram of conventional electrode, the distal GCV was considered to be near the origin of PVC-1. However, radiofrequency (RF) application for the distal GCV (maximum power: 15 W, contact force: 3–21 g, duration: 105 seconds, and generator impedance drop: from 179 to 149 Ω) failed to eliminate the PVC-1, despite subsequent additional RF applications.

**Fig. 3:**
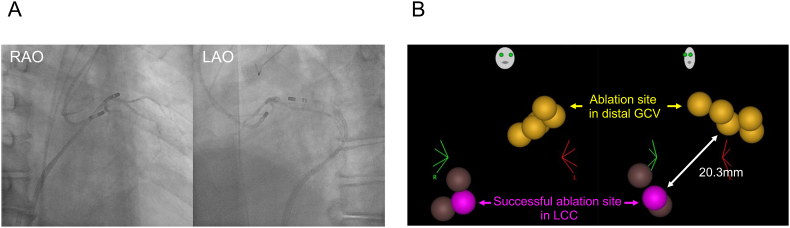
(A)The synthetic fluoroscopic images of the placement of ablation catheter in distal GCV and LCC. (B)The ablation tags in distal GCV and LCC. The minimum distance between distal GCV and successful ablation site in LCC was 20.3mm. RAO, right anterior oblique; LAO, left anterior oblique; GCV, great cardiac vein; LCC, left coronary cusp.

Next, ablation catheter was placed above the LCC via retrograde *trans*-aortic approach (Fig. 2C). Based on the synthetic fluoroscopic image and three-dimensional mapping system (CARTO 3 Version 7.2, Biosense Webster, Diamond Bar, CA, USA), the LCC was anatomically adjacent to distal GCV with a distance of 20.3 mm (Fig. 3A and B). In PVC-1, the electrograms of ablation catheter preceded the PVC-QRS onset by 13 ms both in conventional and microelectrodes (Fig. 2D). In PVC-2, the electrograms preceded the PVC-QRS onset by 32 ms both in conventional and microelectrodes. Pace-mapping at distal electrodes of ablation catheter was good for PVC-2 with a score of 92 % (Fig. 2D). Because the LCC was considered to be close to the origin of PVC-2, RF application was delivered for the LCC to eliminate PVC-2. Notably, the initial RF application for the LCC (maximum power: 35 W, contact force: 13–51 g, duration: 49 seconds, and generator impedance drop: from 107 to 101 Ω) unexpectedly resulted in the simultaneous elimination of both PVC-1 and PVC-2. Additional RF applications were performed at the successful ablation site to ensure durable PVC suppression. No PVC recurrence was observed for 9 months after the ablation procedure.

## 3 Discussion

**Fig. 4:**
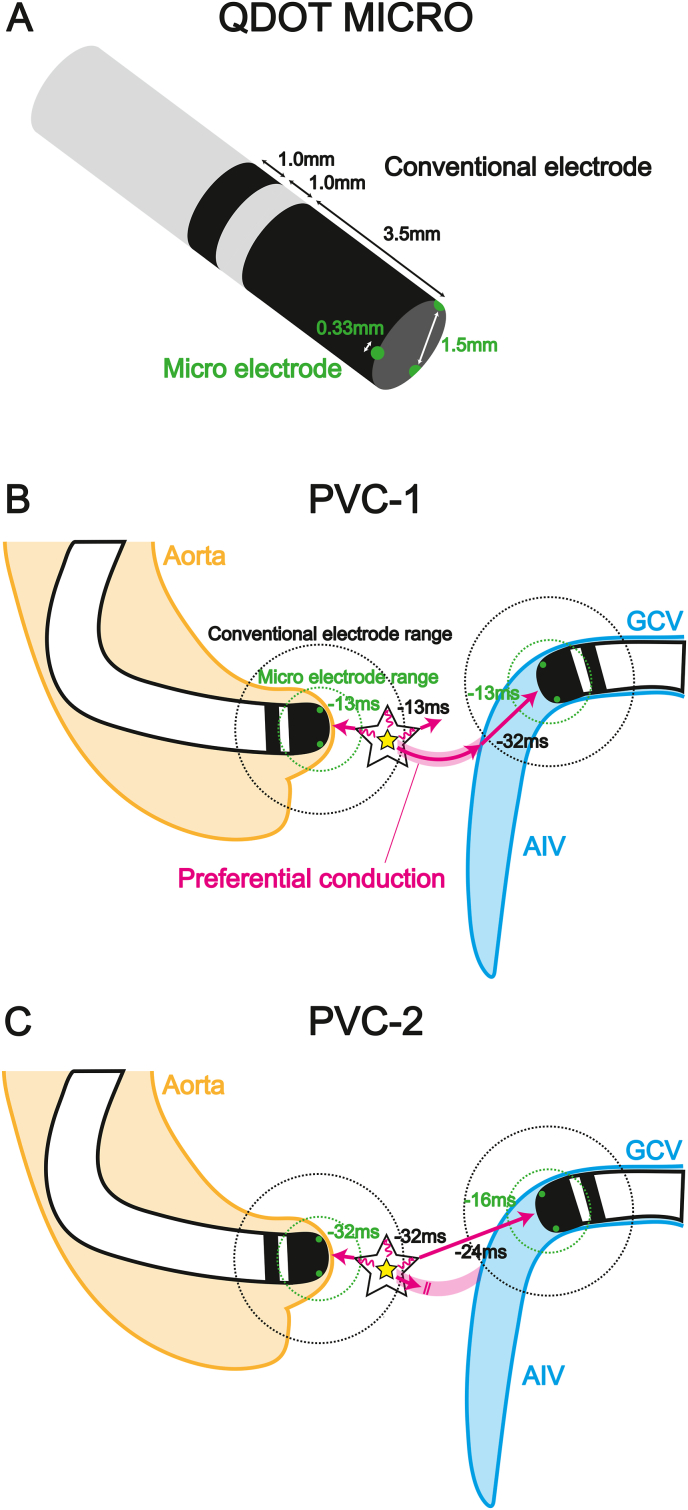
(A) Schematic of QDOT MICRO ablation catheter. The Conventional electrode has 3.5 mm electrode length and 1.0 mm interelectrode. The three microelectrodes have 0.33 mm electrode length and 1.5 mm interelectrode spacing. (B) Hypothesized mechanism of PVC-1. The origin was surrounded by a barrier disturbing rapid conduction to the adjacent myocardium near the LCC. In contrast, the excitation rapidly conducted to the AIV/GCV through a preferential conduction pathway between the origin and the vicinity of AIV/GCV. (C) Hypothesized mechanism of PVC-2. The preferential conduction pathway between the origin and vicinity of AIV/GCV was blocked, resulting in centrifugal propagation through the barrier surrounding the origin. PVC, premature ventricular contraction; GCV, great cardiac vein; AIV, anterior interventricular vein; LCC, left coronary cusp.

The QDOT MICRO ablation catheter has three microelectrodes with 0.33 mm electrode length and 1.5 mm interelectrode spacing, in addition to the conventional electrode with 3.5 mm electrode length and 1.0 mm interelectrode spacing (Fig. 4A). Although the interelectrode spacing was similar, the electrode length was far larger in conventional electrode than in microelectrodes, making conventional electrode was likely to contain far-field potential [2]. Regarding catheter ablation for PVC, even if the bipolar electrograms of the conventional electrode markedly precede the PVC-QRS onset, origin of PVC is not always close to the tip of ablation catheter. In previous report about catheter ablation for PVCs originating from the RVOT, only 36 % of patients showed elimination of PVCs, despite the local electrograms of the conventional electrode preceding the PVC-QRS onset by greater than 23 ms [3]. This is presumably because the conventional electrode detects excitation of PVC origin in the deep endocardium. In contrast, the micro electrode reflects near-field potential which is very close to the tip of ablation catheter, suggesting that the precedence of the bipolar electrograms of the micro electrode may be associated with superficial origin and successful elimination of PVC. Regarding ablation mode, the QDOT MICRO catheter has two different ablation setting; QMODE+ and QMODE. QMODE+ is a very high-power short-duration ablation setting dedicated to pulmonary vein isolation (power: 51–90 W, duration: 1–4 seconds, irrigation flow: 8 ml/minute). QMODE is a conventional ablation setting (power: 1–50 W, duration: no limit, irrigation flow: 4–15 ml/minute). In the present case, we used QMODE for all RF application.

In the present case, while PVC-1 demonstrated good pace-mapping and great electrogram precedence in the conventional electrode in the distal GCV, suggesting that the origin of PVC-1 seemed to be close to the distal GCV (Fig. 2B), its ablation success occurred unexpectedly in the LCC. The QDOT MICRO ablation catheter can measure surface temperature. The output of RF application and irrigation flow are controlled according to the surface temperature. This feature may result in reduced output in tissues with low blood flow, such as coronary sinus, and deep lesions may not form. In the present case, the lesion of RF application was deeper in the LCC than in the distal GCV. Actually, in the previous study, PVCs originating near the AIV/GCV are reported to be eliminated from the adjacent left coronary cusp or LV endocardium when the distance between the AIV/GCV and ablation site is less than 13.5 mm [4], but the distance between the distal GCV and success point in the LCC was 20.3 mm in the present case (Fig. 3B). In addition, in all PVCs originating neat the GCV with successful endocardial ablation, the differences of precedence between the GCV and endocardial success site are reported to be less than 7 ms [5]. However, the difference of precedence between the GCV and the LCC was 32–13 = 19 ms in the present case (Fig. 2). Accordingly, we assumed that if the origin of PVC-1 had been near the GCV, the LCC would have been too far away to eliminate PVC-1. In the LCC, the electrograms of the conventional electrode preceded the PVC-QRS onset only by 13 ms, but the degree of precedence coincided with that of the microelectrodes. We hypothesize that the origin of PVC-1 was close to the LCC, but the true origin potential was too tiny to be detected by an ablation catheter, and the origin was surrounded by a barrier disturbing rapid conduction to the adjacent myocardium near the LCC, resulting in delayed conduction to the LCC. In contrast, preferential conduction pathway between the origin and the vicinity of AIV/GCV allowed rapid propagation, leading to the earliest activation in proximal AIV and distal GCV (Fig. 4B). If the origin of PVC-1 had been near the AIV/GCV, the electrograms of conventional electrode of ablation catheter in the LCC would have preceded those of the microelectrodes because wave front would have propagated toward the LCC from the origin of PVC-1 near the distal GCV. However, the electrograms in both electrodes preceded the PVC-QRS onset by only 13 ms in LCC, supporting the origin of PVC-1 was close to the LCC.

Regarding PVC-2, pace-mapping was good in the LCC, and the electrograms in conventional electrodes of ablation catheter preceded the PVC-QRS onset by 32 ms in the LCC, compared to 24 ms in the distal GCV (Fig. 2B–D). These findings indicated the origin of PVC-2 was near the LCC, and we decided to deliver RF application for the LCC. Given that PVC-1 and PVC-2 were simultaneously eliminated by RF application in the LCC, their origins were the same or closely located. The reason why PVC-2 showed different QRS morphology and different early activation site from PVC-1 was presumably because the preferential conduction pathway between the origin and vicinity of AIV/GCV was blocked, resulting in centrifugal propagation through the barrier surrounding the origin (Fig. 4C). This explanation was consistent with the observation that PVC-2 had a longer coupling interval than PVC-1.

## 4 Conclusion

The coincident precedence of electrograms in both conventional and microelectrodes may be associated with superficial origin and successful PVC elimination.

## Funding statement

This research did not receive any specific grant from funding agencies in the public, commercial, or not-for-profit sectors.

## Ethical statement

**Patient consent statement:** The patient provided consent for publication.

## Declaration of competing interest

The authors declare that they have no known competing financial interests or personal relationships that could have appeared to influence the work reported in this paper.
